# Supplementary figures and images for: Characterization of atypical Ebola virus disease in ferrets
Source: PLoS Pathog. 2026 May 4;22(5):e1013916. doi: 10.1371/journal.ppat.1013916 (PMC13155671; doi:10.1371/journal.ppat.1013916)

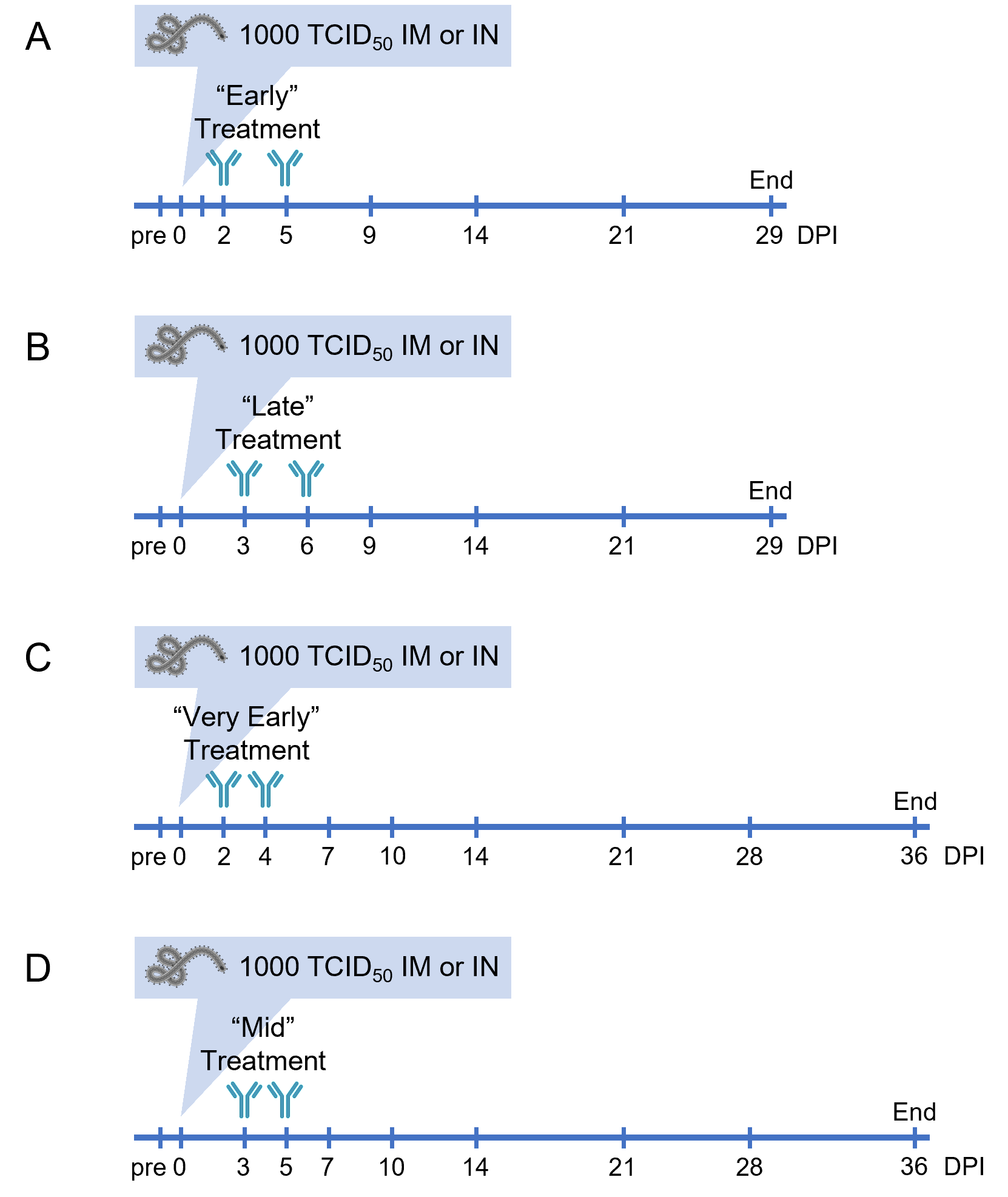

Supplement: S1 Fig — mAb efficacy evaluation was performed over two independent experiments, with the “early” and “late” antibody treatments tested in one experiment (A, B) and the “very early” and “mid” treatments tested in a second experiment (C, D). All animals were inoculated on 0 days post-infection (DPI) with a target dose of 1000 TCID50 EBOV via either the IN or the IM route, after which they were treated with 30 mg/kg each of antibody on 2 and 5 DPI (“early”) (A), 3 and 6 DPI (“late”) (B), 2 and 4 DPI (“very early”) (C), or 3 and 5 DPI (“mid”) (D). Blood and swab samples were collected from all animals on all days indicated on the schematic. The first experiment ended on 29 DPI (A, B), while the second experiment ended on 36 DPI (C, D). Created, in part, in BioRender. Banadyga, L. (2026) https://BioRender.com/ fa8wlyn. (TIF) [file ppat.1013916.s001.tif]

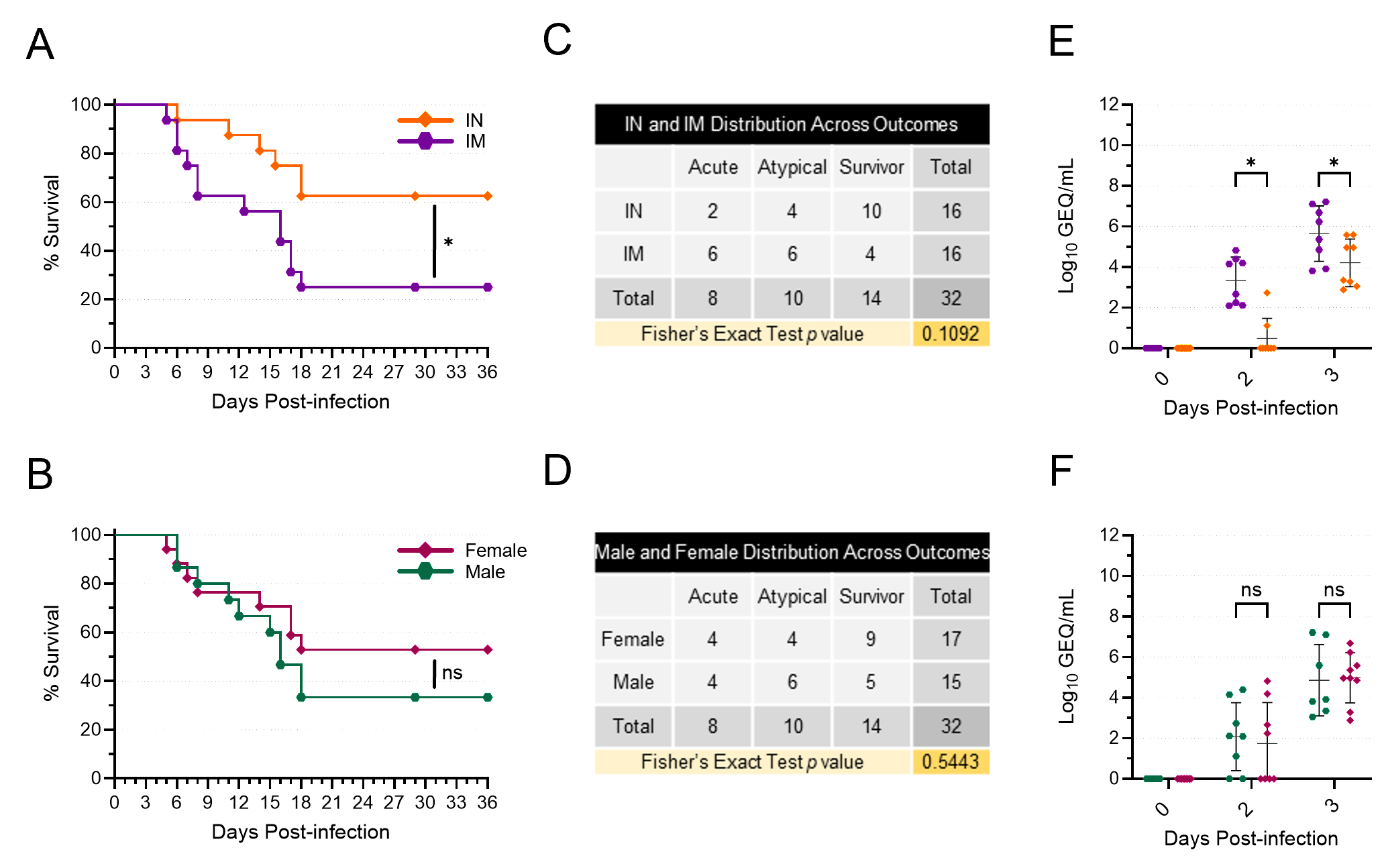

Supplement: S2 Fig — To determine whether route of inoculation or the sex of the animals affected survival (A, B), disease outcome (C, D), or virus replication (E, F), ferret data was re-analyzed for all mAb-treated animals (n = 32). Comparing animals inoculated via the intranasal (IN) route (n = 16) versus the intramuscular (IM) route (n = 16), regardless of the treatment they received, revealed a statistically significant difference in their survival curves using the Logrank (Mantel-Cox) test (A), suggesting that IN-inoculated animals were less likely to succumb to EBOV infection. Analysis of the hazard ratios indicated that IM-inoculated animals exhibited a ~ 3-fold higher risk of death than IN-inoculated animals, consistent with earlier mortality in IM-inoculated animals compared to IN-inoculated animals. No significant association between inoculation route and disease outcome (i.e., Acute, Atypical, or Survivor) was identified using Fisher’s exact test, although IN-inoculated animals skewed towards survival (C). Comparing the mean levels of viral RNA within the first three days of infection (i.e., before mAb treatments were administered) revealed significantly higher levels in IM-inoculated animals compared to IN-inoculated animals at both days 2 and 3 post-infection using unpaired t-tests (Holm-Sidak) (E). Comparing all male (n = 15) and female (n = 17) animals, regardless of the treatment they received, revealed no statistical difference in their survival curves using the Logrank (Mantel-Cox) test (B). Disease outcome was also not associated with sex using Fisher’s exact test (D). No statistical differences in mean viral RNA levels were identified within the first three days of infection using unpaired t-tests (Holm-Sidak) (F). The vertical area shaded grey, from 12 to 18 days post-infection, represents the window in which atypical disease was observed (A, B). Viral RNA loads, expressed in genome equivalents (GEQ) per millilitre, are indicated by a dot for each animal, with means [file ppat.1013916.s002.tif]

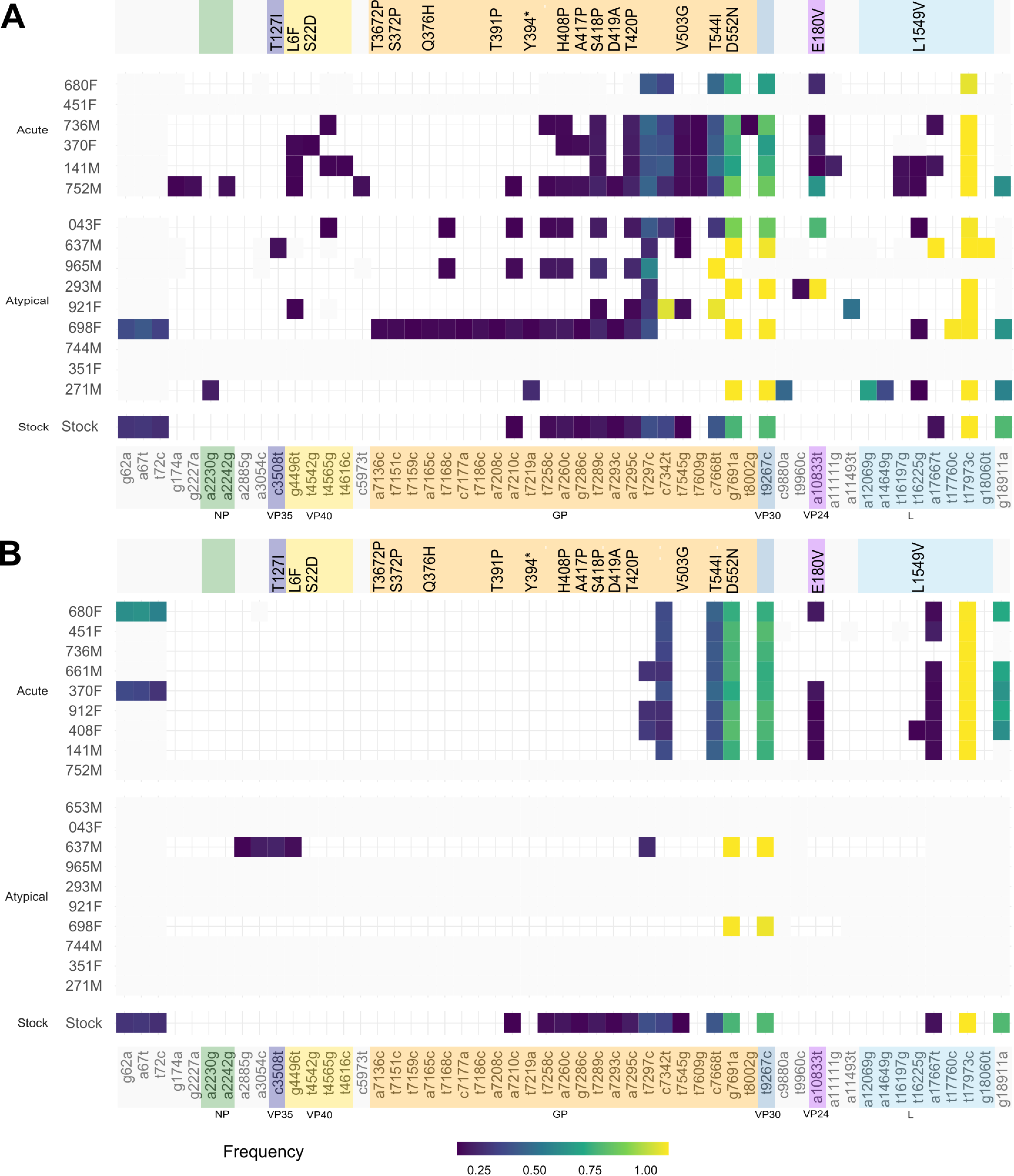

Supplement: S3 Fig — Viral RNA was isolated from brain (A) and liver (B) tissue samples for each of the indicated animals and subjected to next generation sequencing. The frequency of each identified mutation (compared to the EBOV/Mak-C07 reference sequence, Gen Bank Accession No. KJ660347.2) is indicated by a coloured rectangle on the heatmap, with animal ID on the y-axis and genomic mutations on the x-axis. Nucleotide changes are defined across the bottom of each heatmap, using their position in the genome and lowercase lettering for the mutated base pairs. Corresponding amino acid changes are defined across the top of each heatmap, using the amino acid number and the uppercase, single-letter codes for the amino acids. Grey rectangles indicate that no sequencing data was obtained at this position; crossed lines indicate no difference compared to the reference sequence. Sequence data from the stock virus, which was used to inoculate the ferrets, is provided for comparison. (TIF) [file ppat.1013916.s003.tif]

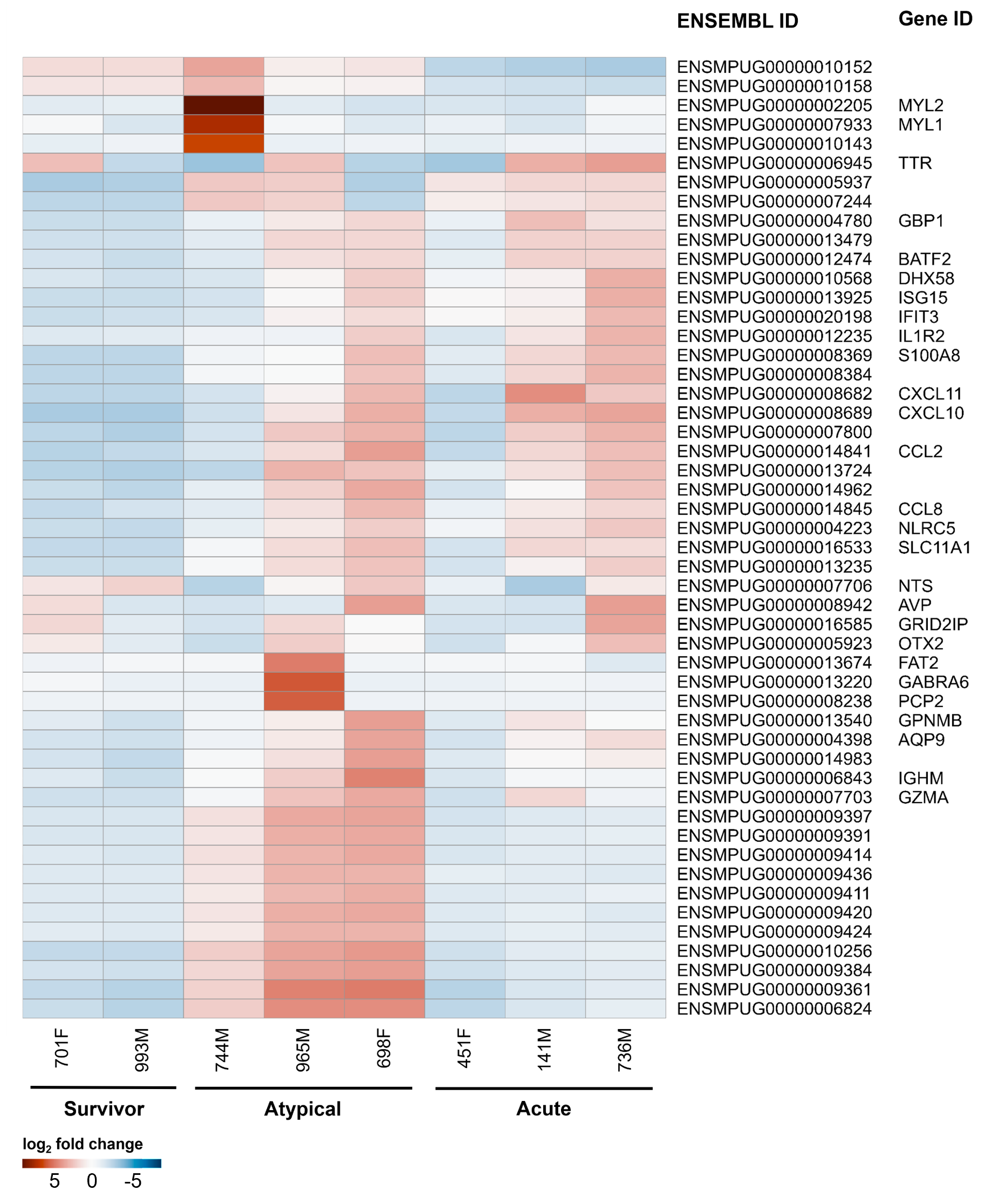

Supplement: S4 Fig — Brain tissue samples from a subset of ferrets (all from the second challenge experiment) were subjected to transcriptomic analysis. A heatmap shows the differential expression of the fifty genes with the most variable expression, including those without names or known functions, shown as log2 fold change centered for each gene. (TIF) [file ppat.1013916.s004.tif]

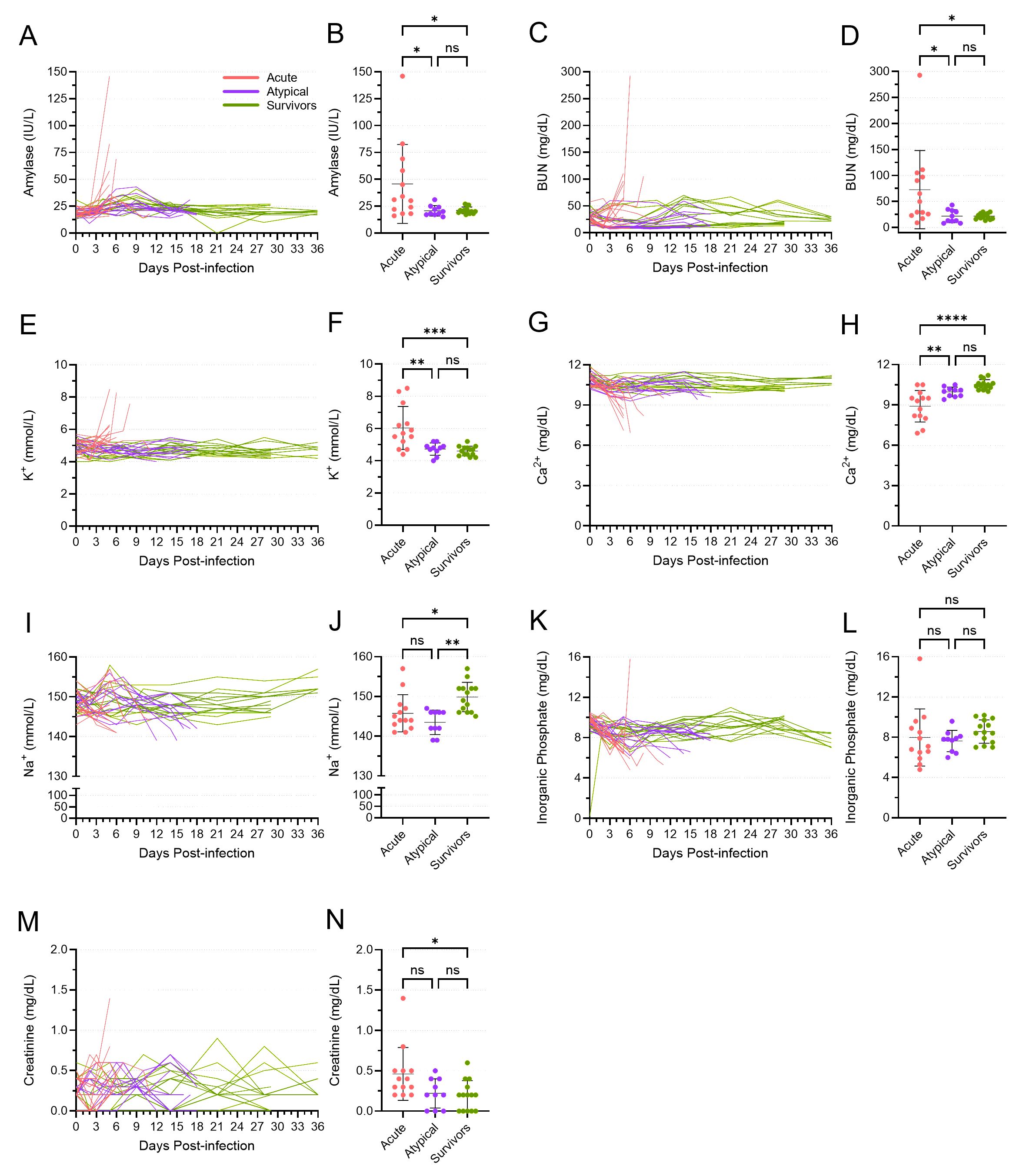

Supplement: S5 Fig — Levels of amylase (A, B), blood urea nitrogen (BUN) (C,D), K+ (E, F), Ca2+ (G, H), Na+ (I, J), inorganic phosphate (K, L), and creatinine (M, N) were quantified in all blood samples from all animals. Levels of each of these analytes are depicted over time (A, C, E, G, I, K, M). The vertical area shaded grey, from 12 to 18 DPI, represents the window in which atypical disease was observed, while the horizontal area shaded grey represents the normal range for each parameter. The levels of each analyte at the terminal time points are also depicted separately (B, D, F, H, J, L, N), with means and standard deviations indicated. Mean levels were compared using a one-way ANOVA with Tukey’s multiple comparison test. ns, not significant; *, p ≤ 0.05; **, p ≤ 0.01; ***, p ≤ 0.001; ****, p ≤ 0.0001. (TIF) [file ppat.1013916.s005.tif]

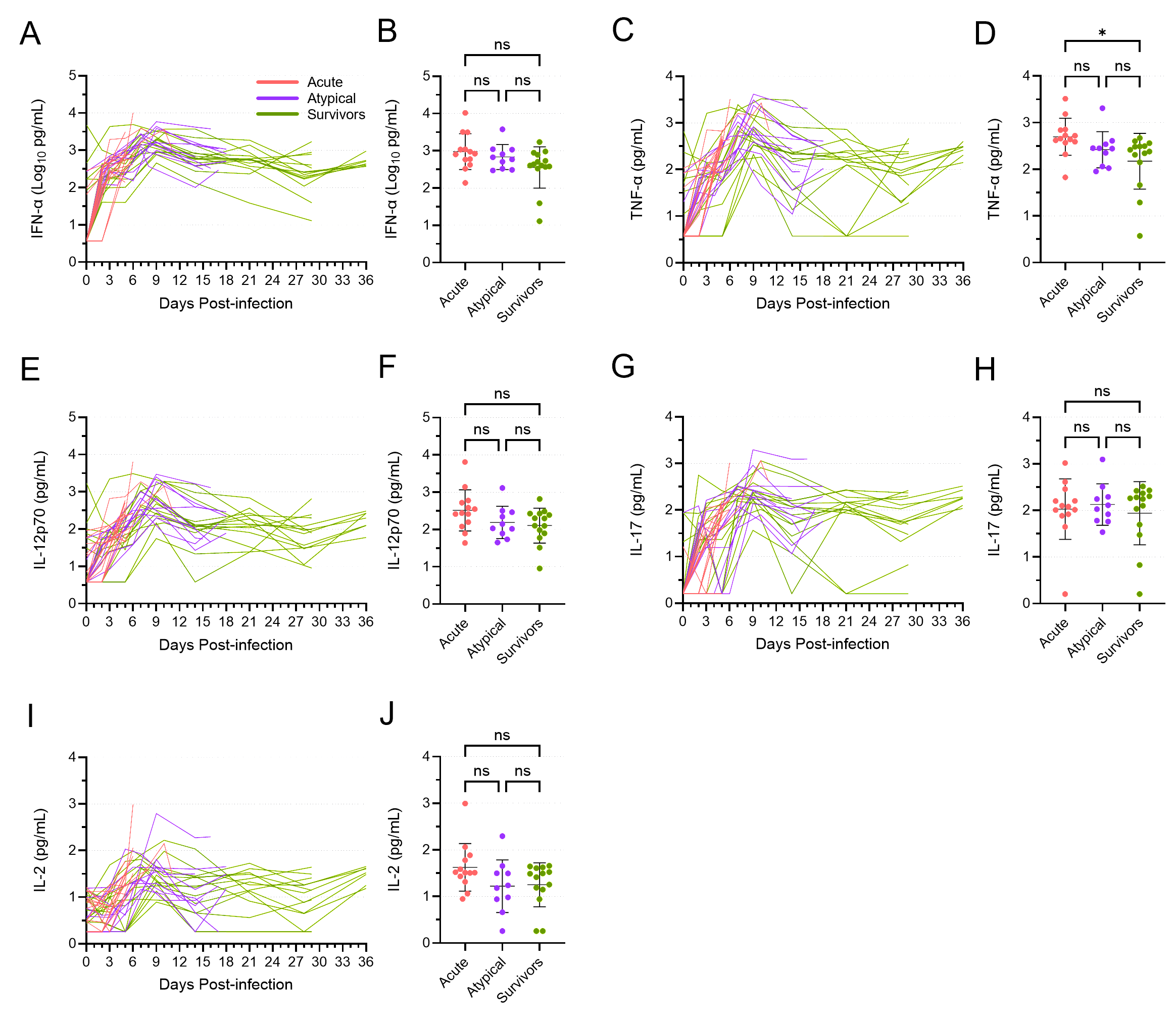

Supplement: S6 Fig — Levels of IFN-α (A, B), TNF-α (C, D), IL-12p70 (E, F), IL-17 (G, H), and IL-2 (I, J) were quantified in all serum samples from all animals via Luminex assay. Levels of each cytokine are depicted over time (A, C, E, G, I). The vertical area shaded grey, from 12 to 18 DPI, represents the window in which atypical disease was observed. The levels of each cytokine at the terminal time points are also depicted separately (B, D, F, H J), with means and standard deviations indicated. Mean levels were compared using a one-way ANOVA with Tukey’s multiple comparison test. ns, not significant; *, p ≤ 0.05. (TIF) [file ppat.1013916.s006.tif]

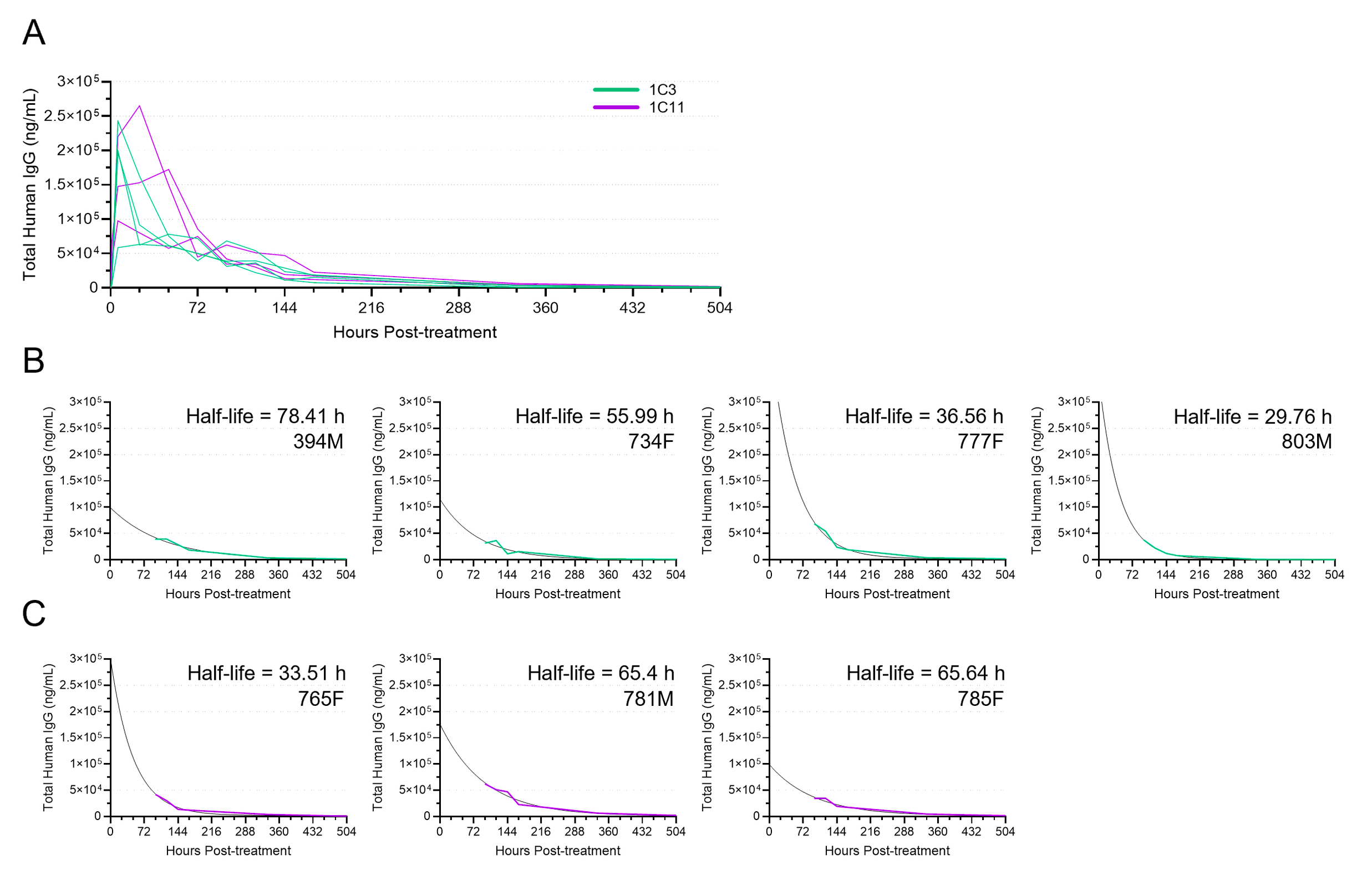

Supplement: S7 Fig — Ferrets were injected intraperitoneally with 30 mg/kg of either 1C3 (n = 4) or 1C11 (n = 4), after which serum samples were collected at 1, 6, 24, 48, 72, 96, 120, 144, 168, 336, and 504 hours post-treatment. Total human IgG (i.e., 1C3 or 1C11) concentrations were quantified in each serum sample via ELISA and depicted over time for each animal (A). Note that human antibodies could not be detected at any time point in one animal injected with 1C11; for this reason, data from this animal were excluded from the analysis. Antibody half-lives were calculated for each animal injected with 1C3 (B) or 1C11 (C) based on the data from 96 to 504 hours using a one-phase decay model to better capture the slow elimination phase. The animal ID is provided for each decay curve, with the sex of the animal indicated (M, male; F, female). (TIF) [file ppat.1013916.s007.tif]
