# Supplementary material for: Characterization of atypical Ebola virus disease in ferrets
Source: PLoS Pathog. 2026 May 4;22(5):e1013916. doi: 10.1371/journal.ppat.1013916 (PMC13155671; doi:10.1371/journal.ppat.1013916)
Supplement: S1 Table — (DOCX) [file ppat.1013916.s008.docx]

| **S1 Table. Summary of Experiments #1 and #2** | | | | | | | | | |
| --- | --- | --- | --- | --- | --- | --- | --- | --- | --- |
| Exp. #​ | Inoculum​ | Inoculation Route​ | Treatment ​ | Treatment Time​ (DPI) | % Survival​ | Animal ID​ | Sex​ | Endpoint (DPI) | Outcome​ |
| 1​ | 1000 TCID_50_ EBOV | IM​ | PBS​ | 2, 5​ | 0%​ | 262 | M​ | 5​ | Acute |
|  |  |  |  |  |  | 301​ | F​ | 5​ | Acute |
|  |  |  | 30 mg/kg each 1C3/1C11 ​ | 2, 5​ | 25%​ | 293​ | M​ | 16​ | ​Atypical |
|  |  |  |  |  |  | 661​ | M​ | 6​ | Acute​ |
|  |  |  |  |  |  | 491​ | F​ | 29​ | Survivor |
|  |  |  |  |  |  | 921​ | F​ | 17​ | ​Atypical |
|  |  |  |  | 3, 6​ | 0%​ | 271​ | M​ | 18​ | Atypical​ |
|  |  |  |  |  |  | 653​ | M​ | 12​ | ​Atypical |
|  |  |  |  |  |  | 912​ | F​ | 7​ | Acute |
|  |  |  |  |  |  | 408​ | F​ | 8​ | Acute |
|  |  | IN​ | PBS​ | 2, 5​ | 0%​ | 297​ | M​ | 6​ | Acute |
|  |  |  |  |  |  | 366​ | F​ | 6​ | Acute |
|  |  |  | 30 mg/kg each 1C3/1C11​ | 2, 5​ | 100%​ | 645​ | M​ | 29​ | ​Survivor |
|  |  |  |  |  |  | 670​ | M​ | 29​ | ​Survivor |
|  |  |  |  |  |  | 939​ | F​ | 29​ | ​Survivor |
|  |  |  |  |  |  | 343​ | F​ | 29​ | ​Survivor |
|  |  |  |  | 3, 6​ | 50%​ | 637​ | M​ | 15​ | ​Atypical |
|  |  |  |  |  |  | 629​ | M​ | 29​ | Survivor​ |
|  |  |  |  |  |  | 351​ | F​ | 18​ | ​Atypical |
|  |  |  |  |  |  | 416​ | F​ | 29​ | Survivor​ |
| 2​ | 1000 TCID_50_ EBOV | IM​ | 30 mg/kg each 1C3/1C11​ | 2, 4​ | 50%​ | 993​ | M​ | 36​ | ​Survivor |
|  |  |  |  |  |  | 965​ | M​ | 16​ | ​Atypical |
|  |  |  |  |  |  | 108​ | F​ | 36​ | ​Survivor |
|  |  |  |  |  |  | 698​ | F​ | 17​ | ​Atypical |
|  |  |  |  | 3, 5​ | 25%​ | 736​ | M​ | 6​ | Acute​ |
|  |  |  |  |  |  | 141​ | M​ | 8​ | Acute |
|  |  |  |  |  |  | 977​ | F​ | 36​ | ​Survivor |
|  |  |  |  |  |  | 451​ | F​ | 5​ | Acute​ |
|  |  | IN​ | PBS​ | 2, 4 | 0%​ | 680​ | F​ | 5​ | Acute |
|  |  |  | 30 mg/kg each 1C3/1C11​ | 2, 4 | 75%​ | 198​ | M​ | 36​ | ​Survivor |
|  |  |  |  |  |  | 744​ | M​ | 18​ | ​Atypical |
|  |  |  |  |  |  | 701​ | F​ | 36​ | ​Survivor |
|  |  |  |  |  |  | 388​ | F​ | 36​ | ​Survivor |
|  |  |  |  | 3, 5​ | 25%​ | 752​ | M​ | 11​ | ​Acute |
|  |  |  |  |  |  | 345​ | F​ | 36​ | ​Survivor |
|  |  |  |  |  |  | 043​ | F​ | 14​ | ​Atypical |
|  |  |  |  |  |  | 370​ | F​ | 6​ | Acute​ |
